# The Effects of Laboratory Contamination of Implant Abutment Screw and Connection on Reverse Torque Value – An In Vitro Study

**DOI:** 10.1002/cre2.70222

**Published:** 2025-09-16

**Authors:** Hamed Bahrami Maleki, Mona Bazooband, Parviz Amini

**Affiliations:** ^1^ Department of Prosthodontics, School of Dentistry Ilam University of Medical Sciences Ilam Iran; ^2^ General Dentist Shiraz Iran; ^3^ Department of Prosthodontics, School of Dentistry Kerman University of Medical Sciences Kerman Iran

**Keywords:** implant abutment contamination, reverse torque value, screw loosening, screw preload

## Abstract

**Objectives:**

This in vitro experimental study aimed to evaluate the effects of laboratory contamination of implant abutment screws and connection surfaces on reverse torque values (RTVs), as an indicator of screw preload loss.

**Material and Methods:**

Forty‐five Dentis One Q implants and 45 CCM UCLA abutments were randomly assigned into three groups (*n* = 15 per group). Group 1 (control) involved uncontaminated abutments and screws with no restorations. Group 2 (screw contamination) used new abutments attached with screws contaminated by laboratory materials (porcelain powder, metal debris, and polishing paste). Group 3 (connection contamination) included screw‐retained restorations fabricated under contaminated conditions and attached using new screws. All samples were subjected to standardized torque (250 N·cm), thermocycling (1500 cycles between 5°C and 55°C), and subsequent RTV measurement. One‐way ANOVA and Tukey's post hoc tests were used for statistical analysis (α = 0.05).

**Results:**

Mean RTVs (SD) were 218 (0.15) N·cm (control), 181 (0.14) N·cm (screw contamination), and 207 (0.11) N·cm (connection contamination). RTVs in the screw contamination group were significantly lower than both the control (*p* < 0.001) and connection contamination groups (*p* < 0.001). The difference between the control and connection contamination groups was not statistically significant (*p* = 0.08).

**Conclusions:**

Laboratory contamination of implant components can significantly reduce reverse torque values, particularly in cases of screw contamination, indicating an increased risk of screw loosening. Contamination control during prosthetic procedures is essential to maintaining implant stability.

## 1 Introduction

Despite the significant success rate of dental implants, biological and prosthetic complications remain common (Morena et al. 2024; Amini et al. 2022). One of the most frequent mechanical complications is the loosening of the abutment screw, which can compromise the stability and longevity of implant‐supported prostheses (Bahrami et al. 2020; Liang et al. 2021). The primary function of the screw in implant procedures is to generate sufficient clamping force to connect the two components, maximizing stability at the implant‐abutment interface (Kim et al. 2023). Incomplete or ineffective tightening of the screw can result in its loosening or fracture, leading to clinical failures. Lee et al. (2020). reported that screw loosening occurs in 0.2% to 45% of cases, with single crowns being particularly vulnerable.

Screw loosening is multifactorial and may result from inadequate preload, poor implant positioning or occlusion, mismatched or misaligned components, excessive occlusal forces, or suboptimal screw design (Linden et al. 2018; Abraham et al. 2022). Clinically, this may cause prosthesis misfit, instability, or even fracture, affecting both function and patient satisfaction (Back et al. 2024).

When torque is applied to the abutment screw, it generates contact forces between the screw head and abutment, as well as between the screw threads and the implant's internal threads (Rathe et al. 2021). These forces produce tension (preload) in the screw, causing elastic elongation (Shinohara et al. 2019; Wang et al. 2009). Upon release, elastic recovery leads to clamping forces, which are essential for maintaining the mechanical integrity of the connection (Yao et al. 2023). A higher preload generally correlates with greater resistance to loosening (Tsuruta et al. 2018).

During various clinical and laboratory procedures—such as abutment preparation, wax‐up, casting, ceramic layering, glazing, and clinical try‐ins—implant components are repeatedly handled, increasing the risk of contamination (Adawi et al. 2023).

The three selected contaminants in this study—porcelain powder, metal debris, and polishing paste—were specifically chosen because they are commonly produced during these routine steps. Their solid consistency and direct contact with implant surfaces make them highly relevant for assessing potential mechanical issues, such as reduced preload and screw loosening.

However, while previous studies have emphasized biological contamination (e.g., saliva or blood), limited data exist on the impact of solid, laboratory‐induced contaminants. Moreover, the specific effects of such contamination on either the screw itself or the internal connection—when exposed separately—have not been comparatively investigated.

Recent studies have shown that interface contamination, even in trace amounts, can alter preload values and increase the risk of mechanical failure under cyclic loads. Additionally, implant macrogeometry and abutment design influence screw stability and reverse torque values (Gehrke et al. 2023; Gehrke et al. 2023). These findings suggest that solid contaminants may significantly compromise the mechanical integrity of implant systems, especially under thermal cycling, which mimics intraoral temperature variations.

To the best of our knowledge, no previous study has directly compared the effects of screw contamination versus connection contamination using clinically relevant, laboratory‐derived solid contaminants. Furthermore, the preload loss following thermocycling in these two contamination scenarios remains unexplored.

Therefore, the aim of this study was to evaluate the effect of laboratory‐induced contamination of either the abutment screw or the connection surface on reverse torque values, simulating realistic prosthetic procedures and thermal cycling conditions.

## 2 Materials and Methods

**Figure 1:**
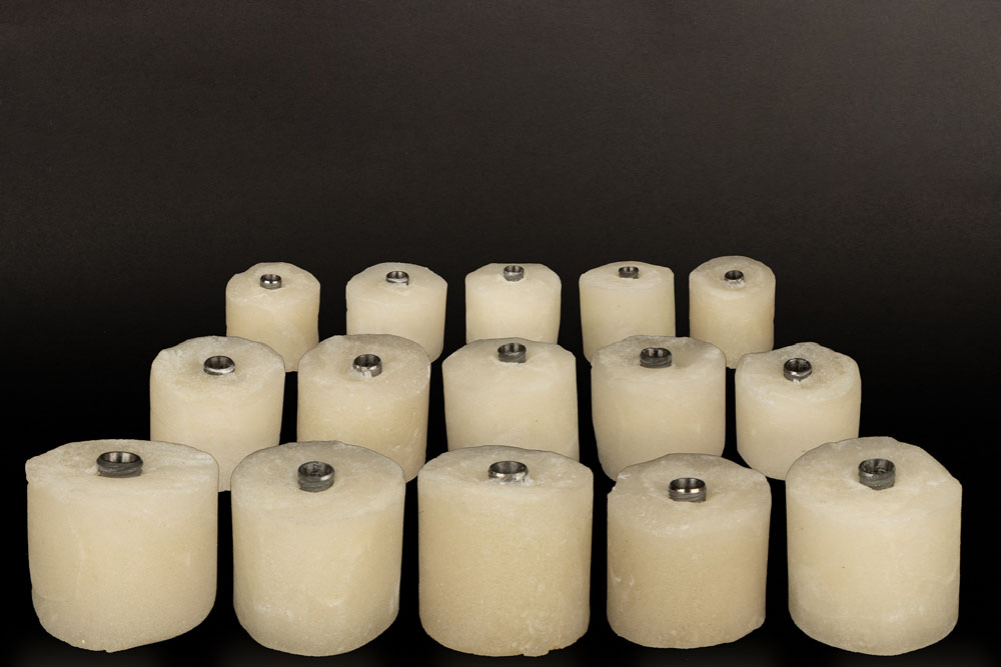
Fixtures were placed in resin blocks.

The study was conducted using experimental and laboratory methods, involving a total of 45 Dentis One Q fixtures (4 × 10 mm, Grade 4, Titanium; Dentis Co. Ltd. Daegu, South Korea), and 45 CCM (Castable Customizing Material) UCLA Dentis abutments (Dentis Co. Ltd. Daegu, South Korea). The fixtures were embedded in resin blocks (Meliodent Rapid Repair; Kulzer GmbH, Hanau, Germany) up to the first thread at a vertical 90° angle and secured with fixators (Figure 1).

Samples were randomly assigned to three groups (*n* = 15 per group) using a computer‐generated randomization table. The study groups were divided based on the condition of the abutments and screws.

**Figure 2:**
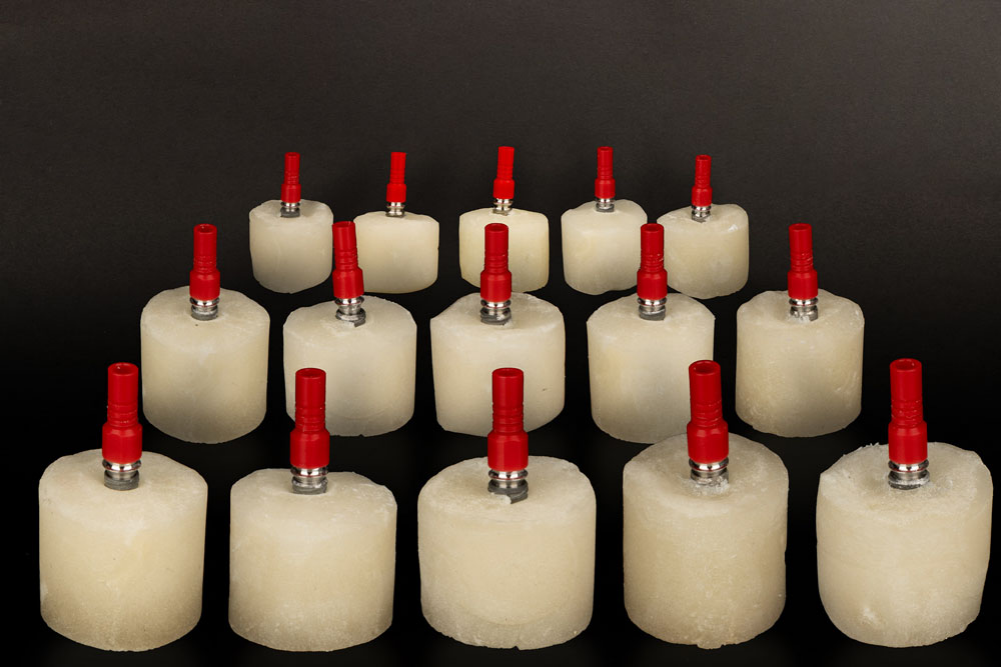
CCM Abutments were placed on the fixtures.

The control group (Group 1) consisted of 15 CCM UCLA abutments, each attached to a fixture using a new screw, with no restorations fabricated or attached (Figure 2).

**Figure 3:**
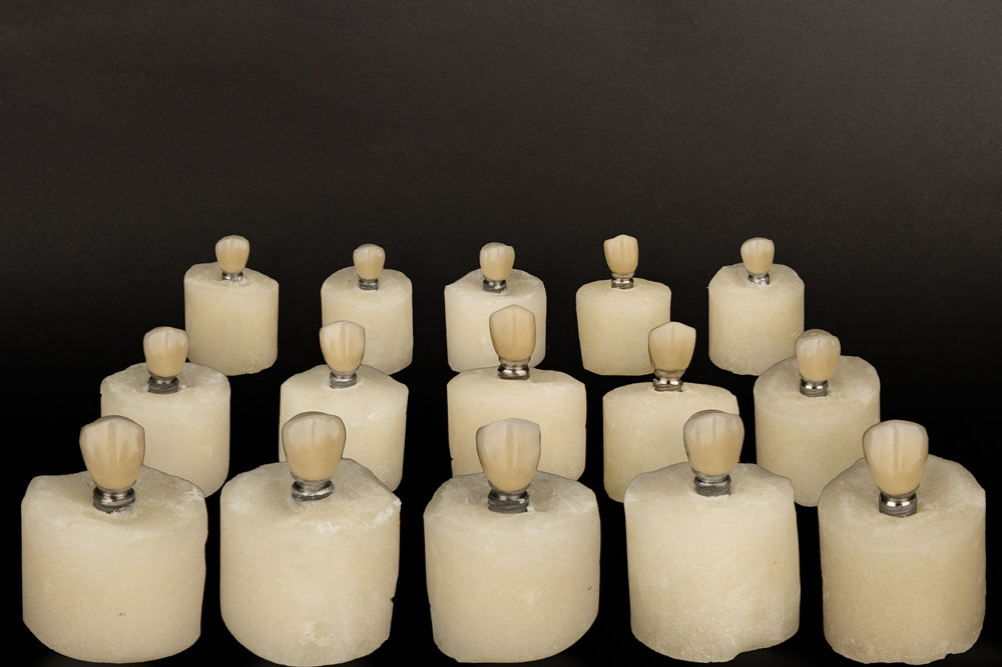
Restorations made by the laboratory.

A total of 15 CCM UCLA abutments and their corresponding screws were selected for the fabrication of 15 screw‐retained restorations. To simulate contamination, a laboratory‐simulated mixture of 0.05 mg was prepared by weight, composed of porcelain powder (VITA VMK Master; VITA Zahnfabrik, Bad Säckingen, Germany), metal debris (Kera Cobalt‐Chrome; Kennametal Dental, Germany), and polishing paste (Universal Polishing Paste; Ivoclar Vivadent, Schaan, Liechtenstein). This mixture was applied evenly using a microbrush to ensure consistent and standardized contamination across all samples. All 15 restorations were fabricated following a standardized laboratory protocol, which included the following steps: (1) wax‐up to design the restoration on the abutments; (2) selective cut‐back to create space for porcelain layering; (3) casting of the wax patterns using a suitable dental alloy—a cobalt‐chromium base metal alloy (Kera Cobalt‐Chrome; Kennametal Dental, Germany)—to form the metal substructures; (4) porcelain application and sintering in a high‐temperature furnace; and (5) glazing to achieve a smooth, esthetic final surface (Korkmaz 2024). The entire fabrication process was carried out under the contaminated conditions described above. After the restorations were completed, the screws were removed (Figure 3).

For Group 2 (screw contamination): The previously contaminated screws were used to attach new, uncontaminated abutments—without any restorations—to the implant fixtures.

For Group 3 (connection contamination): The screw‐retained restorations—with contaminated abutment connections—were attached to the implants using new, uncontaminated screws.

No cleaning or decontamination procedure was performed in either group before the final torque application.

To reduce bias, the operator performing the reverse torque measurements was blinded to the group allocations.

For all groups, an initial torque pressure of 250 N·cm was applied using a digital torque meter (Imada HTGS series, Imada Co. Ltd. Toyohashi, Japan). After 10 min, a final torque pressure of 250 N·cm was reapplied. All samples underwent 1500 thermal cycles between 5°C and 55°C, each lasting 60 s to simulate oral temperature fluctuations (Al‐Zordk et al. 2021).

**Figure 4:**
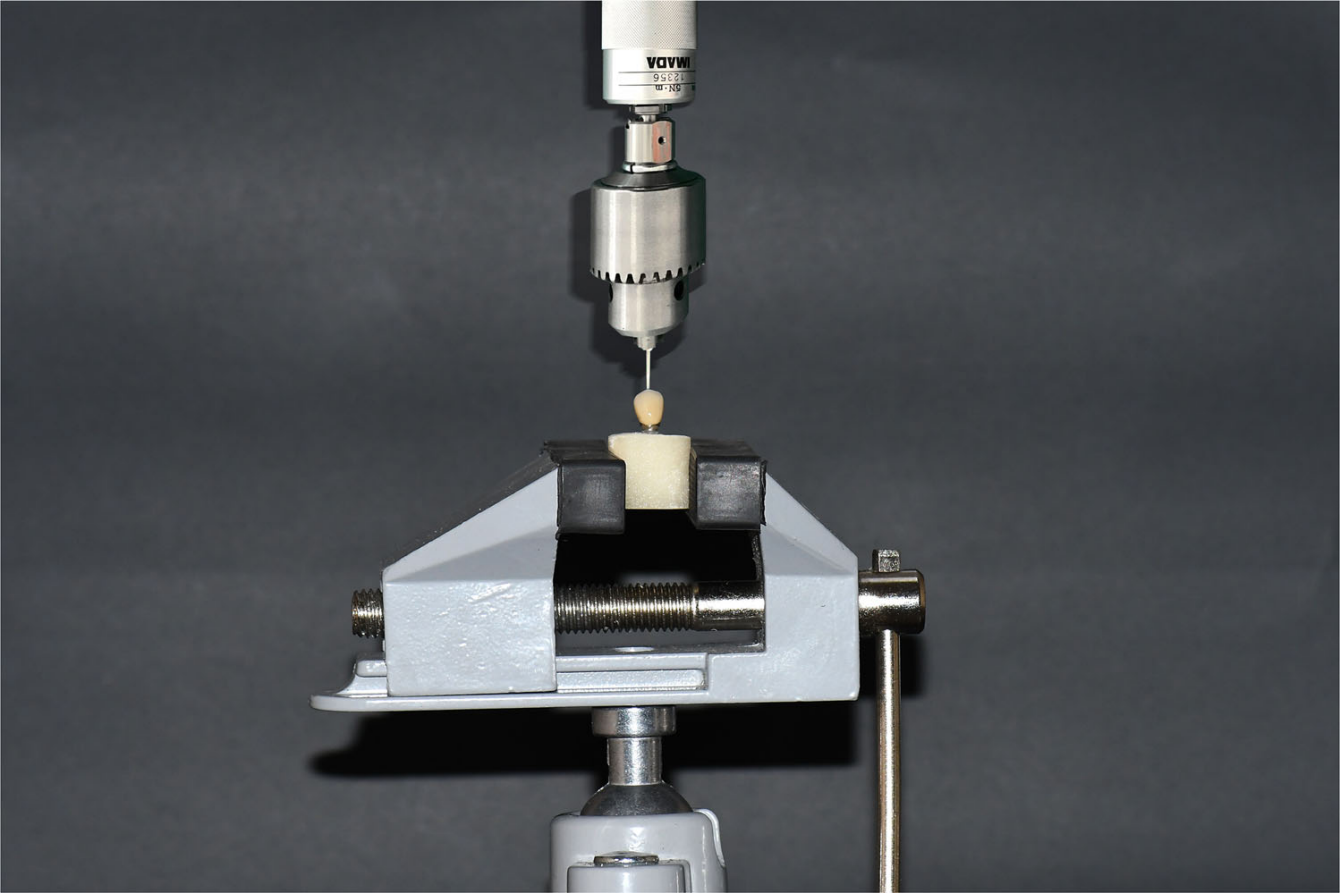
Specimen under digital torque meter for measuring torque.

Reverse torque values (RTVs) were recorded for each specimen using the same digital torque meter. Torque loss (i.e., the difference between the applied torque and the measured RTV) was calculated as an indicator of preload loss (Figure 4).

A priori sample size estimation was performed using G*Power (version 3.1), assuming an effect size of 0.4, power of 80%, and α = 0.05, resulting in a required sample size of 15 per group. The normality of data distribution was assessed using the Shapiro–Wilk test.

All statistical analyses were conducted using SPSS software (version 28.0; IBM, Armonk, NY, USA). A one‐way analysis of variance (ANOVA) was used to determine whether there were statistically significant differences in RTVs among the three groups. A significance threshold of *p* < 0.05 was set for all analyses. If ANOVA results were significant, Tukey's post hoc test was employed to perform pairwise comparisons between groups.

## 3 Results

**Figure 5:**
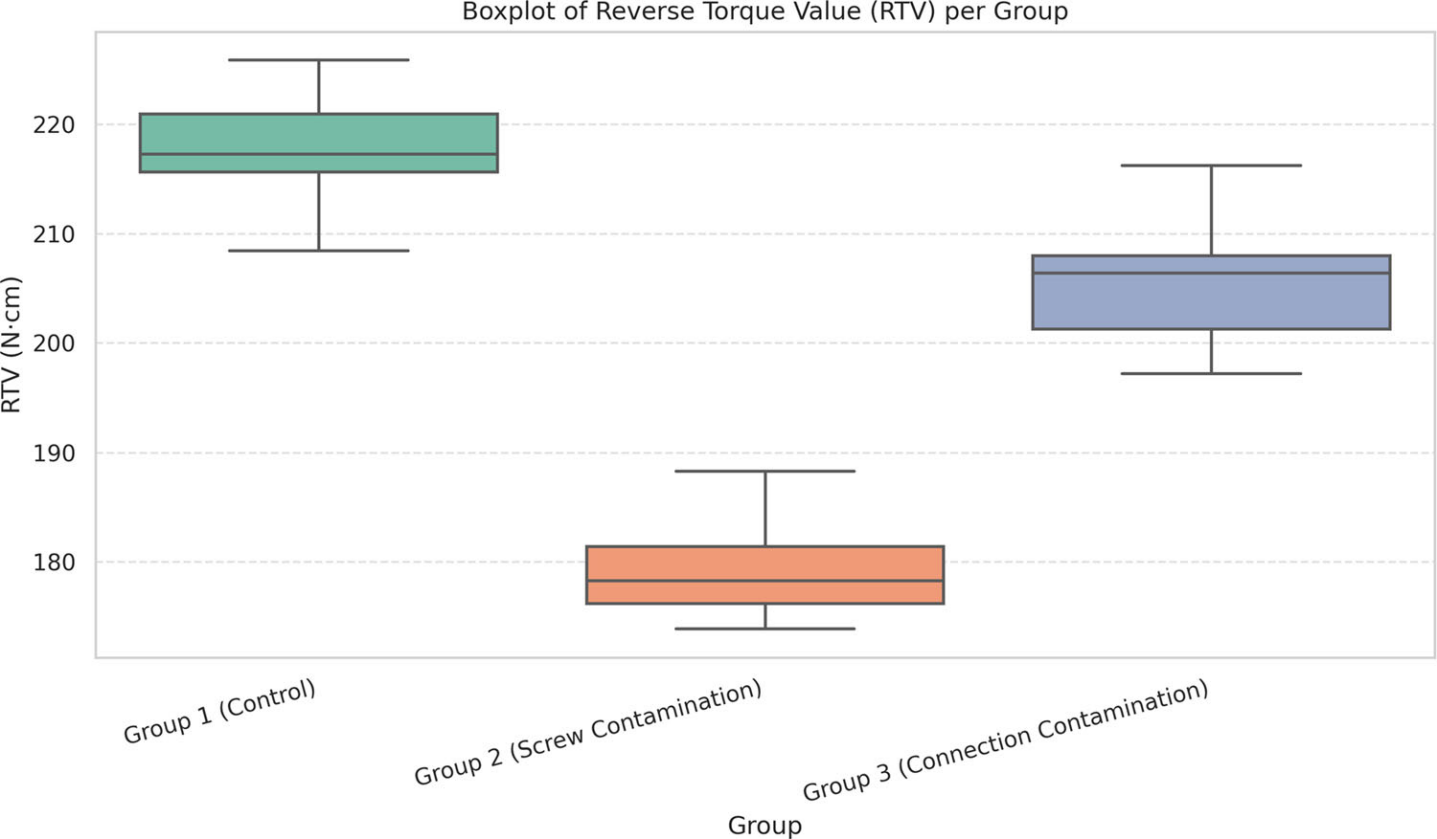
distribution of RTV values across the three groups, highlighting the differences in spread and central tendency.

The raw data for each group (15 samples per group) are reported in Table 1. According to the study results, the mean (standard deviation) of the reverse torque values was 218 (0.15) N·cm in the control group, 181 (0.14) N·cm in the screw contamination group, and 207 (0.11) N·cm in the connection contamination group (Table 2). Figure 5 shows the distribution of RTV values in all three groups, highlighting the variations in data spread and central tendency.

**Table 1:** Raw data of all groups (*n* = 15 per group).

Group 1 (control group) (N·cm)	Group 2 (Screw Contamination) (N·cm)	Group 3 (Connection Contamination) (N·cm)
0.182	0.189	0.222
0.208	0.193	0.196
0.227	0.175	0.202
0.230	0.201	0.218
0.229	0.159	0.188
0.224	0.178	0.215
0.228	0.164	0.212
0.198	0.190	0.204
0.233	0.183	0.219
0.212	0.168	0.201
0.221	0.189	0.210
0.217	0.192	0.198
0.237	0.184	0.218
0.215	0.188	0190
0.202	0.154	0.208

**Table 2:** Mean ± standard deviation of reverse torque values (N·cm) in the control, screw contamination, and connection contamination groups.

Group	Mean RTV (N·cm)	SD	95% Confidence interval		Minimum RTV (N·cm)	Maximum RTV (N·cm)
			Lower bound	Upper bound		
Group 1 (Control group)	218	0.15	209	226	182	237
Group 2 (Screw contamination)	181	0.14	173	188	154	201
Group 3 (Connection contamination)	207	0.11	201	213	188	222

**Figure 6:**
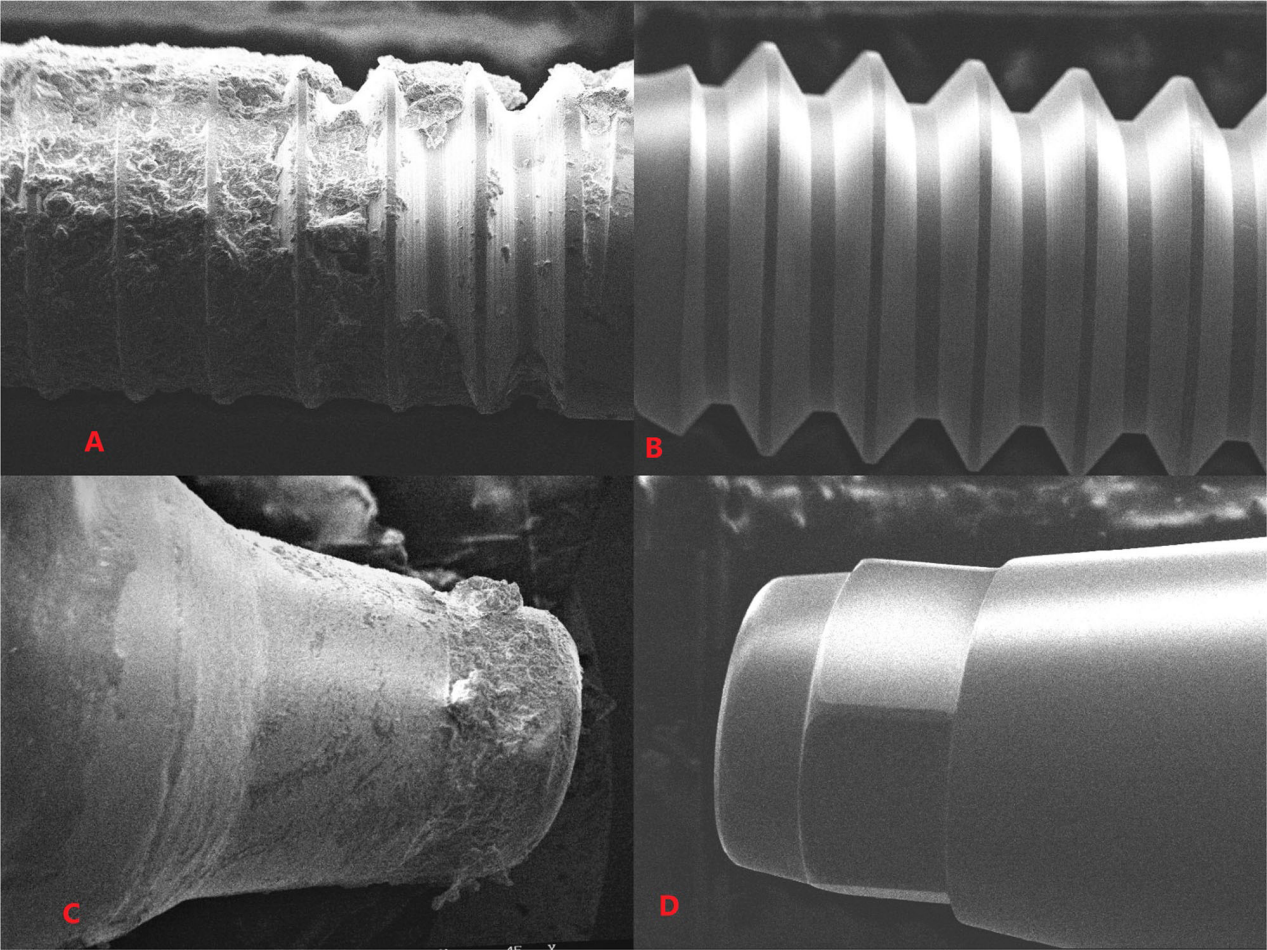
Scanning Electron Microscope (SEM) images of implant abutment screws and connections. (A) Contaminated abutment screw, (B) Uncontaminated abutment screw (magnification: ×70, Scale bar: 100 µm). (C) Contaminated abutment connection and (D) uncontaminated abutment connection (magnification: ×50, Scale bar: 200 µm). Surface debris is evident in the contaminated samples, indicating exposure to polishing and laboratory materials.

SEM analysis confirmed the presence of surface contaminants on the abutment screws and implant connections. As illustrated in Figure 6, images A and C (representing the contaminated screw and connection, respectively) show clear evidence of debris accumulation, surface irregularities, and residual particles, consistent with laboratory contamination. In contrast, images B and D (uncontaminated samples) display smooth and clean surfaces with no visible signs of contamination.

Before conducting inferential statistical analysis, the data distribution was assessed for normality using the Shapiro–Wilk test, which confirmed that the data were normally distributed in all groups (*p* > 0.05). A one‐way ANOVA was then performed and revealed statistically significant differences in reverse torque values among the three groups (*p* < 0.001). Tukey's post hoc pairwise comparisons showed that the differences between the control group and the screw contamination group (*p* < 0.001), as well as between the screw contamination group and the connection contamination group (*p* < 0.001), were statistically significant. However, no statistically significant difference was found between the control group and the connection contamination group (*p* = 0.08). These findings indicate that while screw contamination significantly affects torque values, connection contamination does not lead to a statistically significant reduction compared to the control group.

Table 3 presents a statistical summary of the ANOVA and post hoc comparisons, including p‐values and confidence intervals for each pairwise comparison. This table facilitates clearer interpretation of the statistical relationships among the three experimental groups.

**Table 3:** Statistical summary of one‐way ANOVA and post hoc analyses. The table includes *p*‐values and 95% confidence intervals for pairwise comparisons among the three experimental groups.

Comparison	Mean difference (N·cm)	95% CI (Lower–Upper)	*p*‐value	Significance
Control versus screw contamination	37.0	36.8–37.2	< 0.001	Yes
Screw contamination versus connection contamination.	26.0	25.8–26.2	< 0.001	Yes
Control versus connection contamination	11.0	10.8–11.2	0.08	No

The torque loss values were calculated by subtracting the measured reverse torque values from the initially applied torque of 250 N·cm. In the control group, the torque loss was 32 (0.15) N·cm (250−218). In the screw contamination group, the torque loss was significantly higher at 69 (0.14) N·cm (250−181). In the connection contamination group, the torque loss was 43 (0.11) N·cm (250−207). Although the connection contamination group exhibited greater torque loss than the control, this difference was not statistically significant (*p* = 0.08).

## 4 Discussion

This study investigated the effects of laboratory contamination on reverse torque forces in implant abutment screws and connections. The results revealed a significant reduction in reverse torque forces in contaminated screws and connections compared to new screws, with the most pronounced effects observed in screws contaminated with metal debris and porcelain residues. These findings are consistent with prior research indicating the detrimental effects of contamination on preload forces. For instance, Micarelli et al. (Micarelli et al. 2013). reported a 15%–25% reduction in reverse torque forces for screws contaminated with solid debris, such as titanium particles, which impair mechanical stability. Similarly, Lee et al. (2015). highlighted the significant impact of titanium debris due to the settling effect, which occurs as surface irregularities are smoothed under pressure during screw tightening. The settling effect refers to the initial loss of preload caused by microscopic adjustments at the screw–abutment interface. As these micro‐irregularities flatten under static or functional pressure, clamping force decreases, explaining part of the observed torque loss in contaminated samples.

The mechanisms underlying this torque loss involve several interrelated physical and mechanical processes. Solid contaminants such as metal and porcelain debris create surface irregularities at the screw–abutment–implant interface. These irregularities interfere with optimal screw seating and reduce the true contact area, thereby decreasing frictional resistance and clamping force—both essential for maintaining preload. Moreover, these particles can act as spacers, preventing full tightening of the screw and leading to a lower initial preload. During tightening or functional loading, part of this preload is further lost due to the settling effect. Additionally, the abrasive nature of these contaminants may cause microdamage or wear at the contact surfaces, potentially leading to permanent degradation of the mechanical integrity of the joint and reduced reverse torque values.

However, this study also diverges from some previous findings. Mostafavi et al. (2021). and Koosha et al. (2020). reported variable effects of liquid contaminants—such as saliva, blood, and chlorhexidine—on reverse torque forces, with some contaminants, such as chlorhexidine, even increasing reverse torque. These discrepancies may be attributed to the differing physical and chemical properties of solid versus liquid contaminants. Solid contaminants disrupt surface smoothness, leading to preload loss, whereas liquid contaminants may alter adhesion or lubrication at the interface. This distinction underscores the importance of understanding the specific nature of contaminants when assessing their impact on implant screw performance.

The reduction in reverse torque forces observed in this study can be attributed to multiple factors. Metal and porcelain debris compromise the smoothness of the screw–abutment–implant interface, reducing the clamping and frictional forces required to maintain preload. Additionally, the settling effect further exacerbates preload loss, as described in earlier studies (Abraham et al. 2022; Varvara et al. 2020). This effect, caused by pressure‐induced smoothing of coarse points on the screw and abutment surfaces, typically dissipates 2%–10% of the initial preload, thereby reducing the torque required to loosen the screw.

Importantly, these mechanisms highlight the potentially irreversible impact of solid debris on the integrity of the screw–abutment interface, even in controlled laboratory settings. The current study's focus on solid contaminants—such as metal particles and porcelain powders—represents a novel contribution to the existing body of literature. While previous research has predominantly examined the effects of liquid contaminants, this study addresses a relatively understudied yet clinically relevant category of contaminants frequently encountered during laboratory procedures (Mar et al. 2023; Kermanshah et al. 2020). By highlighting the mechanical and practical implications of metal and porcelain debris, this study provides valuable insights for improving contamination control practices. The findings are particularly relevant for clinicians and dental technicians, emphasizing the need for meticulous protocols during wax‐up, casting, porcelain application, and glazing to reduce the risk of contamination and ensure the long‐term stability of implant‐supported prostheses.

This study also aligns with and reinforces prior observations by Adawi et al. (2023) and Gumus et al. (2014), who confirmed that contamination—particularly with blood—significantly reduces reverse torque forces and increases the risk of screw loosening. These studies demonstrated that blood contamination forms protein layers and biofilm on titanium surfaces, impairing mechanical stability. Similarly, our findings revealed that other types of contamination, including metal and porcelain debris, can likewise reduce reverse torque forces and potentially compromise the stability of implant connections.

Furthermore, recent evidence emphasizes that contamination at the implant–abutment interface may not only compromise mechanical integrity but also biological sealing. A study published in 2023 evaluated bacterial leakage in Morse taper implant–abutment connections using the DNA–DNA checkerboard hybridization technique and demonstrated the potential for microbial infiltration despite precise mechanical fit. This highlights the multidimensional risk of contamination, where even microscopic debris may contribute to microgap formation, facilitating bacterial colonization and subsequent peri‐implant diseases. Although our study focused primarily on mechanical consequences, these microbial findings emphasize the broader clinical risks of contamination, further supporting the need for stringent contamination control at all procedural stages (Teixeira et al. 2023).

While cleaning methods such as argon plasma and ultrasonic cleaning have demonstrated effectiveness in removing contaminants (Hofmann et al. 2023; Tokarz et al. 2023), Shemtov‐Yona et al. (2024) reported that reverse torque forces in cleaned screws remain lower than those in new screws, even after cleaning. This suggests that certain contaminations may cause microdamage or alter the surface properties of the screw–abutment interface in ways that are not fully reversible, even with advanced cleaning methods. This further highlights the importance of proactive prevention during both laboratory and clinical procedures.

Despite these findings, some clinical questions remain underexplored. For example, the feasibility of reusing contaminated screws after decontamination, and whether standardized cleaning protocols can restore their mechanical integrity, remains uncertain. Future studies should directly compare new and cleaned screws using both mechanical and surface analysis methods to address this clinical concern.

Conflicting findings in the literature regarding screw loosening may arise from inconsistencies between clinical and laboratory data. For example, clinical follow‐ups have reported screw loosening rates ranging from 0% to 13% (Varvara et al. 2020; Huang and Wang 2019), whereas laboratory studies often demonstrate a higher prevalence. These discrepancies may stem from variations in study designs, testing conditions, or materials used, underscoring the need for standardized laboratory models to improve reproducibility and reliability across studies.

## 5 Limitations

Some limitations for this study should be considered. The experimental design, while providing controlled insights, does not fully replicate clinical scenarios, such as functional loading conditions or dynamic intraoral environments. Moreover, the study did not simulate cyclic or functional loading, which plays a critical role in screw loosening over time in clinical settings. Another limitation is the variability of contaminations encountered in real‐world clinical and laboratory workflows, which may involve complex mixtures not fully replicated in this study. Additionally, challenges exist in implementing contamination control protocols consistently across dental laboratories and clinics, and this study does not evaluate their practicality or cost‐effectiveness. Furthermore, there was no quantitative assessment of the properties of the contaminants—such as particle size, morphology, or concentration—which could influence their mechanical effects.

Future research should address these gaps by exploring advanced cleaning methods, such as argon plasma and ultrasonic techniques, and investigating the long‐term effects of contamination on screw performance under functional loading conditions. Moreover, the development of contamination‐resistant materials and more sophisticated laboratory models would enhance the consistency and clinical relevance of future research.

Taken together, these findings highlight the multifactorial nature of torque loss due to contamination and underscore the necessity of integrating contamination control into routine prosthetic workflows.

Based on the findings, the null hypothesis that contamination does not significantly affect reverse torque forces is rejected. Laboratory contamination, particularly with solid debris, significantly reduces reverse torque forces, with screw contamination having the most pronounced effect. These results emphasize the importance of rigorous contamination control during laboratory and clinical procedures to ensure the stability and longevity of implant‐supported prostheses. Future research focusing on improved cleaning methods and material innovations could further mitigate these challenges.

## 6 Conclusion

Laboratory contamination of implant abutment screws and connection surfaces was found to significantly reduce reverse torque values in this in vitro study, indicating a potential risk for screw loosening. Among the contamination types examined, screw contamination appeared to have a more pronounced effect. However, given the experimental nature of this study, caution is warranted in directly extrapolating these findings to clinical practice. Further in vivo studies are needed to confirm the clinical relevance of these results. Meanwhile, implementing careful contamination control protocols during prosthetic laboratory procedures may help minimize the risk of preload loss and screw instability.

## Author Contributions

Hamed Bahrami Maleki contributed to the study conception, design, supervision, and manuscript drafting. Mona Bazooband performed the laboratory procedures, data collection, and contributed to manuscript revision. Parviz Amini carried out data analysis and assisted in the final editing of the manuscript. All authors reviewed and approved the final version of the manuscript.

## Conflicts of Interest

The authors declare no conflicts of interest.

## Data Availability

All data generated or analyzed during this study are included in this manuscript (see Table 1).
